# Integrating virtual reality into ADHD therapy: advancing clinical evidence and implementation strategies

**DOI:** 10.3389/fpsyt.2025.1591504

**Published:** 2025-04-22

**Authors:** Anithamol Babu, Akhil P. Joseph

**Affiliations:** ^1^ School of Social Work, Marian College Kuttikkanam Autonomous, Kuttikkanam, India; ^2^ School of Social Work, Tata Institute of Social Sciences, Jalukbari, India; ^3^ Department of Sociology & Social Work, Christ (Deemed to be University), Bengaluru, India

**Keywords:** virtual reality therapy, ADHD treatment innovation, neurocognitive interventions, digital therapeutics in psychiatry, executive function rehabilitation, immersive cognitive training

Attention-Deficit/Hyperactivity Disorder (ADHD) remains a persistent and multidimensional challenge in mental health research and clinical practice, manifesting as pervasive deficits in cognitive control, neurocognitive processing, and affective regulation (1, 2). Conventional interventions such as stimulant pharmacotherapy, cognitive-behavioral therapy (CBT), and neurofeedback have demonstrated efficacy in ADHD management. However, they face significant challenges, including technological constraints, limited accessibility, high financial burdens, clinician training disparities, and ethical concerns. These limitations manifest differently across high- and low-resource settings, where disparities in infrastructure, funding, digital literacy, and workforce capacity exacerbate inequities in access to ADHD care. In high-income regions, challenges may stem from regulatory hurdles and fragmented digital integration, whereas in low- and middle-income contexts, fundamental barriers such as inadequate internet connectivity, lack of VR-compatible hardware, and absence of trained professionals severely restrict the feasibility and reach of such interventions surrounding data security and prolonged immersive exposure (3). Adverse effects, including stimulant-induced sleep disturbances and appetite suppression, coupled with inconsistent adherence due to treatment fatigue and systemic barriers such as socioeconomic disparities and inadequate access to specialized ADHD care, underscore the need for alternative and complementary therapeutic approaches (4). In response to these limitations, Virtual Reality (VR) has emerged as a novel therapeutic modality, offering immersive, neuroadaptive interventions designed to enhance attentional control, cognitive flexibility, and self-regulation (5). VR presents an advanced, multimodal therapeutic tool that facilitates immersive cognitive training, fosters neuroadaptive plasticity, and optimizes higher-order attentional control and cognitive flexibility within controlled, ecologically valid settings (5, 6). While VR holds substantial theoretical promise, its integration into ADHD therapy must be grounded in rigorous empirical evidence—a point explored further in subsequent sections to avoid redundancy.

Incorporating VR into ADHD treatment represents a paradigm shift, augmenting traditional modalities through ecologically valid, interactive simulations aimed at cognitive and behavioral remediation (7). VR-based interventions are underpinned by principles such as neuroplasticity, reinforcement learning, and cognitive regulation, cohering with dominant theoretical frameworks like the dual-pathway model, Barkley’s executive dysfunction model, and the cognitive-energetic theory (8). These paradigms offer explanatory value for deficits in inhibitory control, sustained attention, and motivational regulation—domains VR can potentially address through adaptive, immersive tasks (9–11). However, these models have been critically scrutinized for their narrow neurocognitive focus and their neglect of the broader socio-cultural, ecological, and relational contexts in which ADHD symptoms emerge (12). Overemphasis on dysfunction-oriented frameworks may pathologies neurodivergent experiences and obscure systemic, environmental, and identity-based factors that modulate attentional functioning. In response, biopsychosocial paradigms—including the neurodiversity movement—propose an alternative lens through which ADHD is viewed not as a deficit to be corrected, but as a natural variant within the spectrum of cognitive diversity (13). These models prioritize relational and contextual elements, advocating a strengths-based, socially situated understanding of attentional and behavioral differences (14). Incorporating such perspectives into the theoretical grounding of VR enables a more ethical, inclusive, and person-centered approach to intervention design. Rather than relegating VR to a role of neurocognitive correction, this epistemologically pluralistic orientation reframes it as a medium for fostering empowerment, skill acquisition, and environmental fit. This reconceptualization challenges technocratic reductionism, guarding against overly deterministic narratives and promoting interventions that respect neurodivergent identities while addressing functional needs.

However, VR should not be conceptualized as a universal solution; rather, its role must be carefully delineated as an adjunct to conventional therapies rather than a complete paradigm shift in therapeutic intervention. The phenotypic variability in ADHD’s neurocognitive and behavioral manifestations necessitates personalized adaptation of VR interventions, ensuring alignment with specific attentional, impulsivity-related, and emotional regulation deficits (8). Also, VR presents several critical limitations that must be systematically addressed. Sensory overstimulation and cybersickness—discussed further in later sections—can diminish therapeutic engagement, especially among users with high sensory sensitivity (15). In addition to these concerns, immersive environments may paradoxically contribute to attentional fragmentation and executive function disruption in some users. Over-reliance on VR simulations could limit the generalization of learned skills to real-world contexts, potentially reinforcing maladaptive attentional patterns (16). Moreover, ethical and privacy challenges arise from the collection of sensitive behavioral and biometric data in VR settings, necessitating stringent regulatory safeguards to prevent misuse or breaches of patient confidentiality (17–19). Long-term adherence and engagement sustainability also remain uncertain, as the novelty effect may wear off over time, leading to declining participation. Moreover, the scalability of VR in ADHD care is significantly hindered by interrelated challenges: the high cost of equipment, infrastructural limitations in low-resource settings, and the scarcity of adequately trained clinicians. While high-income contexts face challenges related to integration and standardization, in low- and middle-income countries (LMICs), these barriers are further intensified by limited internet access, high data costs, and insufficient institutional funding. For instance, the average cost of VR systems can exceed $4,000 annually, making them inaccessible to most public health facilities. Even reduced-cost alternatives, priced at $1,500, remain out of reach for many community clinics. These infrastructural deficits are compounded by the absence of standardized training programs that would enable clinicians to confidently and ethically apply VR in clinical practice. Addressing these intersecting challenges requires multi-tiered solutions: subsidization strategies, context-sensitive technological adaptation, and investment in clinician education and certification.

Rigorous empirical investigations are necessary to evaluate VR’s safety profile, compare it against conventional interventions across different ADHD subtypes, and develop standardized clinical protocols that mitigate risks while maximizing therapeutic benefits. Several pressing research inquiries must be addressed: How does VR compare with established pharmacological and behavioral interventions in sustaining long-term cognitive and functional outcomes? How do neurophysiological mechanisms underpinning VR’s therapeutic effects compare to those activated by conventional treatments? Can VR interventions be feasibly adapted across developmental stages, ensuring accessibility and sustained adherence? What is the dropout rate in VR-based interventions compared to traditional therapies, and what factors contribute to engagement or attrition? Also, the cognitive and emotional risks of prolonged VR exposure, including sensory overstimulation, emotional dysregulation, and maladaptive digital dependency, require systematic investigation. These questions necessitate methodologically rigorous trials, longitudinal cohort studies, and neurophysiological investigations to establish VR’s empirical legitimacy in ADHD management.

Advancing VR research in ADHD requires a multidisciplinary, evidence-based framework, integrating expertise from mental health sciences, neuroscience, psychology, social work, education, and digital therapeutics to drive empirical validation and implementation strategies. Comparative effectiveness studies must assess how VR-based interventions measure against stimulant medication, CBT, and neurofeedback, ensuring a rigorous evaluation of clinical efficacy, neurophysiological impact, and long-term adherence across diverse populations. In addition to these quantitative approaches, including mixed-methods and cross-cultural research designs is essential to fully understand contextual acceptability, feasibility, and user experience—particularly in low- and middle-income countries (LMICs). Engaging stakeholders such as clinicians, educators, patients, and caregivers through qualitative inquiries and participatory action research can provide culturally grounded insights and enhance the ecological validity of interventions. A phased research and implementation model is warranted, beginning with pilot studies in specialized clinical settings to assess feasibility and acceptability. These should be followed by small-scale randomized controlled trials (RCTs) for initial efficacy testing, ideally designed in collaboration with stakeholders including clinicians, caregivers, individuals with ADHD, and community representatives. Involving these groups in trial design can enhance cultural sensitivity, contextual relevance, and participant acceptability, thereby improving recruitment, adherence, and the practical utility of research findings. To further enhance contextual applicability—particularly in under-resourced settings—mechanisms such as user-centered design (UCD) frameworks and community-based participatory research (CBPR) should be employed. These approaches ensure that the voices of end-users inform all stages of intervention development, from content creation to delivery modalities. Additionally, multi-center trials should be used to evaluate generalizability and scalability across diverse settings. Each phase should be accompanied by iterative feedback loops involving clinicians, technologists, and end-users to ensure ecological validity, therapeutic alignment, and responsiveness to evolving community needs. Implementation pathways should include collaborations with mental health institutions, technology developers, and educational settings, ensuring that VR interventions are seamlessly adapted to real-world therapeutic environments. For example, VR-based attention training modules could be tested in specialized ADHD clinics before expanding to school-based interventions for children and adolescents. Moreover, leveraging telehealth platforms and mobile-based VR applications could improve accessibility for patients in remote or resource-constrained regions, facilitating broader integration within mental healthcare systems. A critical component of successful VR integration is the development of structured, competency-based training programs for clinicians, ensuring they acquire expertise in VR-assisted therapeutic application, real-time symptom monitoring, and ethical considerations. Training models should incorporate hands-on clinical simulations, standardized certification programs, and interdisciplinary collaboration with technologists and mental health professionals to bridge the gap between technological advancements and psychiatric practice, thereby reducing inconsistencies in implementation and enhancing treatment reliability and adherence to standardized therapeutic protocols.

Beyond clinical validation, VR’s wide deployment necessitates a comprehensive framework integrating ethical, infrastructural, and systemic considerations, including deeper engagement with systemic ethical issues such as the commodification of digital mental health, algorithmic bias in VR program design, and the potential for inequitable profit-driven deployment in underserved populations to facilitate its responsible and equitable use in ADHD treatment. A structured approach should involve the development of policy-driven guidelines, regulatory frameworks, and interdisciplinary collaborations to address challenges such as screen exposure regulation, sensory processing thresholds, data security, and patient safety. For instance, national health agencies and mental health professional’s associations should collaborate with technology developers to establish evidence-based clinical standards, ensuring VR interventions align with mental health best practices. Besides, integrating VR into existing telepsychiatry models and digital health platforms can promote accessibility while maintaining stringent monitoring and data protection protocols. Implementing these structured strategies will enable VR to transition from a novel technological innovation to a clinically endorsed, scalable, and ethically responsible intervention for ADHD. Moreover, persistent disparities in technological access, affordability, and implementation feasibility pose significant challenges, particularly in low-resource settings (20). In India and other developing contexts, VR’s scalability depends on developing cost-effective models, mobile-compatible adaptations, and interdisciplinary collaborations to expand accessibility. Overcoming these barriers requires strategic government investment, public-private partnerships, and community-driven digital health initiatives, implemented through targeted funding for research grants, clinical trials, and technological development. Establishing VR-integrated ADHD treatment centers in hospitals and mental health clinics, supported by collaborations between universities, healthcare institutions, and technology companies, can ensure its integration into clinical practice. In low-resource settings, leveraging mobile-based VR interventions and subsidizing VR devices for community clinics can enhance accessibility.

Integrating strategies for cultural adaptation occupies a central position in implementation planning, as these adaptations are crucial for ensuring contextual relevance, therapeutic resonance, and sociocultural validity. This includes localizing VR content to reflect indigenous languages, culturally specific behavioral norms, and regionally salient psychosocial stressors. Employing participatory design methods, such as involving caregivers, community leaders, clinicians, and individuals with ADHD in the co-creation and iterative testing of VR modules, can foster cultural resonance and optimize user engagement. Community engagement is also critical for fostering acceptance and sustainability. Initiatives such as awareness campaigns, school-based information sessions, and collaborations with grassroots health workers can demystify the technology and encourage informed uptake. Such efforts should be coupled with ongoing support systems, including community tech hubs and digital literacy programs. A robust policy framework is essential to govern the ethical deployment of VR. Governments and health regulatory bodies should formulate guidelines addressing immersive duration, age-appropriate usage, data privacy safeguards, and integration into national digital health strategies. Public sector investment and multi-stakeholder coordination can further institutionalize VR within mainstream mental health services. These steps will enable VR-based ADHD interventions to transition from an experimental concept to a validated, scalable, contextually grounded, and ethically sound therapeutic modality.

A critical barrier to effective VR integration lies in the necessity of customizing interventions to address the diverse and multidimensional neurocognitive symptomatology characteristic of ADHD subtypes. Given that predominantly inattentive, hyperactive-impulsive, and combined subtype presentations exhibit distinct cognitive, attentional, and behavioral dysfunctions, VR interventions must be tailored to target subtype-specific impairments (7, 21). To enhance engagement and therapeutic efficacy, future VR applications should integrate Serious Games (SGs) that are structured, goal-oriented, and therapeutically grounded. SGs provide a dynamic platform to train executive function, attentional control, impulse regulation, and emotional modulation through interactive, real-time feedback (22). For instance, individuals with predominantly inattentive ADHD may benefit from VR-based sustained attention and working memory tasks, while those with hyperactive-impulsive ADHD may require interventions focusing on impulse control and behavioral self-regulation. Also, integrating adaptive, real-time feedback mechanisms and personalized progression models into VR platforms could optimize engagement and ensure that interventions evolve according to an individual’s therapeutic needs. However, despite its potential, VR-based interventions face several limitations that require systematic evaluation. Long-term efficacy remains uncertain, as the novelty effect associated with immersive digital interventions may diminish over time, leading to declining participation rates. Besides, high dropout rates in VR-based interventions compared to traditional therapies may raise concerns about engagement sustainability and adherence (23). Sensory overstimulation and cybersickness, which are common in VR settings, could further contribute to reduced adherence, particularly among individuals with increased sensory reactivity and hypersensitivity (5). The lack of standardized VR therapy protocols, including session duration, frequency, and structured activities, limits its applicability across diverse clinical settings and increases variability in treatment outcomes. Without a rigorously validated, interdisciplinary, and ethically grounded framework, VR’s integration into ADHD therapy risks stagnation. To counter this risk, concrete policy and institutional measures are necessary—such as the establishment of national clinical guidelines for digital therapeutics, integration of VR competencies into mental health training curricula, allocation of public health funding for VR research and implementation, and development of partnerships between technology developers and public mental health institutions to ensure equitable and sustainable integration, hindered by methodological inconsistencies, unresolved ethical concerns, and systemic barriers to scalability.

A pressing global research imperative is the rigorous evaluation of VR as an ADHD intervention, requiring phased multi-center clinical trials, cross-cultural longitudinal studies, and advanced neurophysiological investigations. Despite its potential, VR remains an underexamined therapeutic tool in ADHD treatment, necessitating comparative efficacy studies that systematically assess its advantages and limitations relative to stimulant medication, cognitive-behavioral therapy (CBT), and neurofeedback (16). Research must focus on standardizing intervention protocols, establishing regulatory frameworks, and defining clinical applicability criteria to ensure that VR-based therapy is not only effective but also ethically and practically deployable across diverse settings (24). Coordinated global efforts are essential, demanding collaboration among leading psychiatric institutions, mental health organizations, technology developers, and policymakers to generate high-impact empirical evidence that informs global clinical guidelines, regulatory standards, and scalable implementation strategies. International collaboration facilitates equitable knowledge production by enabling the sharing of culturally diverse perspectives, fostering inclusive design, and bridging gaps in clinical expertise and infrastructural capacity. Such partnerships can support capacity building in under-resourced settings through shared training programs, open-source platform development, and context-sensitive pilot initiatives. Furthermore, they enable the cross-cultural validation of VR protocols, ensuring that interventions are adaptable, ethically grounded, and relevant across sociocultural contexts. Addressing key challenges—including long-term efficacy, treatment adherence, accessibility disparities, and ethical considerations such as data privacy and immersive exposure risks—requires methodologically rigorous, interdisciplinary research. Without such urgent scientific inquiry and structured global collaboration, VR will remain a fragmented, experimental innovation rather than a validated, evidence-based, and clinically transformative intervention in ADHD management.
